# Microstructure and Mechanical Properties of Precipitate Strengthened High Entropy Alloy Al_10_Co_25_Cr_8_Fe_15_Ni_36_Ti_6_ with Additions of Hafnium and Molybdenum

**DOI:** 10.3390/e21020169

**Published:** 2019-02-12

**Authors:** Sebastian Haas, Anna M. Manzoni, Fabian Krieg, Uwe Glatzel

**Affiliations:** 1 Metals and Alloys, University Bayreuth, 95440 Bayreuth, Germany; 2 Helmholtz-Zentrum Berlin für Materialien und Energie GmbH, 14109 Berlin, Germany

**Keywords:** HEA, high entropy alloys, compositionally complex alloys, mechanical characterization

## Abstract

High entropy or compositionally complex alloys provide opportunities for optimization towards new high-temperature materials. Improvements in the equiatomic alloy Al_17_Co_17_Cr_17_Cu_17_Fe_17_Ni_17_ (at.%) led to the base alloy for this work with the chemical composition Al_10_Co_25_Cr_8_Fe_15_Ni_36_Ti_6_ (at.%). Characterization of the beneficial particle-strengthened microstructure by scanning electron microscopy (SEM) and observation of good mechanical properties at elevated temperatures arose the need of accomplishing further optimization steps. For this purpose, the refractory metals hafnium and molybdenum were added in small amounts (0.5 and 1.0 at.% respectively) because of their well-known positive effects on mechanical properties of Ni-based superalloys. By correlation of microstructural examinations using SEM with tensile tests in the temperature range of room temperature up to 900 °C, conclusions could be drawn for further optimization steps.

## 1. Introduction

For centuries, constructions with high mechanical requirements have been built using steel or iron-based alloys, while titanium or aluminum have been used for light-weight constructions. In gas turbines nickel- or cobalt-based superalloys are used because of their extremely good mechanical properties and oxidation resistance over long periods of time at high temperatures. The main properties of these alloys are given by their base element, while the addition of other elements in small amounts leads to fine adjustments of a specific behavior [1].

An unconventional approach of alloy design is based not on a main element, but on a chemical composition that exhibits a large number of elements with none of them dominating. In this group one differentiates between single-phase high entropy alloys (HEA) [2] and multiphase compositionally complex alloys (CCA). Multicomponent alloys were examined first by Yeh [2] and Cantor [3]. Investigating the microstructure of alloys with different numbers of elements in equiatomic mixtures, Cantor found the five-component, equiatomic alloy Co_20_Cr_20_Fe_20_Mn_20_Ni_20_ to form a single-phase solid-solution. High configurational entropy, due to the large number of elements and similar concentrations, leads to a decrease in the Gibbs free energy and thus a stabilization of one solid-solution phase if no intermetallic phase with low enthalpy of formation exists [2]. Such a single-phase material was claimed to have outstanding properties, e.g., a good strength-ductility behavior over a wide temperature range, thermal stability, wear resistance and high resistance against oxidation of the material [4].

Most alloys containing at least five elements in near equiatomic composition, however, do not crystallize as a single-phase solid-solution, but rather form intermetallic compounds as secondary phases with even lower Gibbs free energy. This leads to the formation of compositionally complex alloys (CCA). They cover a wide range of chemical compositions, with a high number of occurring phases and therefore a wide perspective in terms of applications. We restricted ourselves to have at least five elements and no element should dominate the composition. In the domain of high-temperature materials a gap between steels (<650 °C) and nickel-based superalloys (>850 °C) might be filled by less cost-intensive compositionally complex alloys. The focus should be on mechanical strength, oxidation resistance, processability and, last but not least, the cost factor. We therefore compared our alloy with commercially available alloys. The Ni-Co-Cr-Mo alloy IN 617 shows high-temperature strength and oxidation resistance, while Alloy 800 H has good creep properties and a quite ductile behavior at temperatures below 600 °C. The base alloy of our work shows a similar melting range and is optimized with respect to the features mentioned above. Possible industrial applications could be: Chemical industry appliances and especially land-based steam turbines as well as parts for gas turbines in sections with lower temperatures (700–800 °C).

The development of CCA for mechanical applications at both room and higher temperature is based on different microstructural approaches (see the review article by Manzoni [5]): Interpenetrating phases lead to a high resistance of the material, and additionally interesting electrical or magnetic properties may appear [6]. One example to strengthen the equiatomic high entropy alloy CoCrFeMnNi is the addition of 2 at.% carbon. This leads to a fcc-microstructure with embedded Cr_23_C_6_ particles and higher strength than for the CoCrFeMnNi alloy. The best strength-ductility relation of this carbide-strengthened alloy can be reached by applying the correct thermomechanical treatment [7]. Another example of particle strengthened CCA is the alloy Al_12.35_Co_17.5_Cr_17.5_Fe_35.15_Ni_17.5_ (in at.%). This microstructure exhibits a bcc-matrix with coherently embedded particles of B2 structure. The cuboidal nanoscale particles lead to high strength at room and high temperatures [8].

These three mentioned approaches show the diversity in designing CCA for mechanical applications, using strengthening mechanisms based on very different microscopic features. Singh et al. [9] examined the six-component, equiatomic alloy AlCoCrCuFeNi, with a focus on solidification behavior, using different techniques to analyze the microstructure. Even a high cooling rate by splat-quenching leads to a phase decomposition and the cast alloy shows a microstructure with several phases of different crystal structures, unlike expected solid-solution stabilization due to a high configurational entropy. Recognizing the difficulties of avoiding phase separation, a transition was made from developing a HEA to optimizing a CCA.

Much effort was put into optimizing alloys with near equiatomic composition of AlCoCrCuFeNi, investigating changes in composition and finding correct heat treatment parameters [10], resulting in the chemical composition Al_10_Co_25_Cr_8_Fe_15_Ni_36_Ti_6_ (in at.%). This alloy composition is the base alloy for this work, exhibiting L1_2_-ordered, coherently embedded precipitates in a fcc-matrix, comparable to nickel-based superalloys. Additionally the base alloy shows another third phase, appearing in a needle-like shape up to a length of 50 µm. These needles are very rich in aluminum (28 at.%), with a reduction of all other elements, especially Fe, Co and Cr. TEM investigations identified this phase to have a Heusler type structure.

A way to improve the mechanical behavior is directional solidification, where grains are oriented in loading direction. As a material itself has quite good creep properties, for example in the case of superalloys, the grain boundaries play a strong role in weakening the material at higher temperatures. Avoiding grain boundaries inclined at 45° by the loading direction, shear stresses along these weaker boundaries can be eliminated and the strength is just defined by the microstructure inside of the longitudinal orientated grains [11].

Nickel-based superalloys provide further ideas to improve the high-temperature properties, due to similarities in microstructure. By the addition of Hf and/or Mo, different approaches for optimization are expected: Molybdenum is supposed to partition to the matrix, to have a solid-solution strengthening effect due to its bigger atomic size and therefore to lead to a distorted lattice, acting as an obstacle for dislocation movement [12]. The positive effect of hafnium is due to a strengthening of grain boundaries: A columnar-grained microstructure for example shows an enhancement of creep ductility and lifetime by the addition of hafnium that strengthens the vicinity of grain boundaries vertical to the load stress [13]. Doherty et al. [14] explain this by Hf participating more likely to the γ′-phase Ni_3_(Al, Hf), leading to a strengthening of it. The grain boundaries are therefore strengthened by the fracture-retarding effect of the interlocking γ′-configuration in this areas [15].

## 2. Materials and Methods

### 2.1. Alloy Preparation

In this work all chemical compositions are given in at.%. The composition of the base alloy Al_10_Co_25_Cr_8_Fe_15_Ni_36_Ti_6_ was changed by adding 0.5 at.% hafnium or 1.0 at.% molybdenum on the expense of aluminum. The added amounts and the reduction of aluminum were identified by simulations using the ThermoCalc software [16] with the database TTNi7 [17]. The base alloy in its original composition is also considered.

All constituents with a purity of 99.99% were cleaned in ethanol using an ultrasonic bath and were then melted in a vacuum induction furnace. The material was distributed randomly in a ceramic crucible in the middle of a water-cooled Cu-coil. After evacuating the chamber twice to a pressure of 5∙10^−4^ mbar it was flooded with argon to prevent the evaporation of elements, especially chromium. The ceramic mold was heated up to a temperature of 1400 °C by a second coil and a graphite receptor, thus the material remained in liquid state after casting. To achieve directionally solidified grains in the [001]-direction, the Bridgman process was used and the mold was withdrawn through a water cooled baffle with a speed of 3 mm/min. The cast rods, with a diameter of 20 mm and a length of about 110 mm, were homogenized for 20 h at 1220 °C in the case of the Mo-containing alloy and at 1140 °C in the case of the Hf-containing alloy to avoid eutectic formation determined by differential scanning calorimetry. Subsequent annealing was performed in two different ways for both alloys, 900 °C/50 h and 950 °C/100 h respectively. After heat treatment the rods cooled down to room temperature in the furnace. To remove the oxide layer the samples were initially sand-blasted and afterwards treated with aqua regia. The rods were cut by electrical discharge machining to obtain samples for microscopic and mechanical characterization.

### 2.2. Microstructural Observations

Flat disks were cut from the rods and cut again in the middle to examine the cross and longitudinal section of the microstructure. These surfaces were embedded in a conductive resin, ground, polished with 1 µm diamond slurry and finally polished chemically. The specimens were etched with a solution of 3 g Mo-acid in 100 mL H_2_O, 100 mL HCl and 100 mL HNO_3_ to achieve a better phase contrast by dissolving γ′- and Heusler type phase. For the examination we used a scanning electron microscope (SEM) Zeiss 1540EsB Cross Beam, operating under an accelerating voltage of 30 kV and using the SE2-detector for imaging. Precipitate size and volume fraction were determined using the classification of the Weka segmentation method [18] in the open source software Fiji [19], based on ImageJ [20,21]. More than 500 particles per state were analyzed.

### 2.3. Mechanical Tests

Electrical discharge machining was employed to obtain flat specimens for high temperature tensile tests. The square cross section of the samples was 1.0 × 1.9 mm^2^ and the gauge length was 8 mm, while the entire length of the sample was 25 mm. The specimens were cut out in such a way that the tensile direction was parallel to the [001]-grain-orientation. Before assembling the specimen, their surfaces were ground and a type-S thermocouple was welded for the regulation of temperature on the lower end of the gauge length. The sample was then attached to the ceramic clamping, the radiant heated furnace was closed and heated up to the desired temperature. Tensile tests were performed with a deformation rate of 0.01 mm/s (corresponding to 1.3 × 10^−3^ 1/s). A load cell and a high-resolution camera were logging about four pairs of values for stress and strain in one second. This lead to engineering stress–strain curves for each test, providing mechanical parameters like ultimate tensile strength (UTS), yield strength (YS) and strain to failure (ε_f_).

## 3. Results and Discussion

### 3.1. Chemical and Microstructural Analysis of Al_10_Co_25_Cr_8_Fe_15_Ni_36_Ti_6_

**Figure 1:**
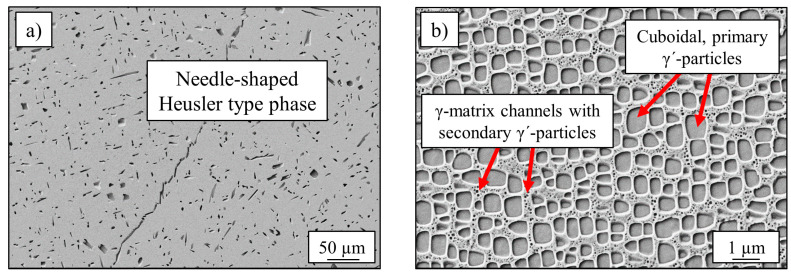
SEM-images of (**a**) Heusler type phase with a needle-like shape and a length up to 50 µm and (**b**) cuboidal γ′-particles with an edge-length up to 400 nm and matrix channels with small and spherical secondary γ′-particles (some 10 nm).

The alloy Al_10_Co_25_Cr_8_Fe_15_Ni_36_Ti_6_ exhibited a dendritic microstructure after the casting process. Dendrites could be dissolved by a homogenization heat-treatment at 1220 °C for 20 h. Subsequent annealing for 50 h at 900 °C lead to a three-phase microstructure: Figure 1a) shows the large (several 10 µm), randomly distributed Heusler type phase, with its characteristic needle-like shape and a volume-fraction of <5%. The γ′-microstructure is displayed in Figure 1b) with a higher magnification with L1_2_-ordered cuboidal shaped γ′-precipitates and small matrix channels with round secondary γ′-particles. The matrix has a face-centered cubic structure [22].

The chemical analyzation of all phases, determined by TEM/EDS, is listed in Table 1 and results in a Co-Fe-Cr rich fcc-matrix and Ni-Al-Ti rich L1_2_-ordered precipitates [10]. The Heusler type phase is very rich in Al, while all other elements are depleted, especially Fe, Co and Cr.

### 3.2. Impact of Bridgman Process on Mechanical Properties

**Figure 2:**
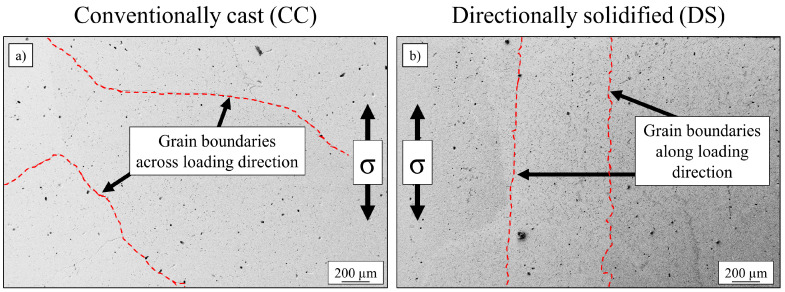
SEM-image of (**a**) conventionally cast, polycrystalline and (**b**) directionally solidified microstructures of the base alloy Al_10_Co_25_Cr_8_Fe_15_Ni_36_Ti_6_ after annealing at 900 °C for 50 h.

An increase of tensile strength was expected by the use of the Bridgman process, resulting in directional solidified samples. The base alloy was cast twice, conventionally cast with randomly orientated grains and directionally solidified, as described in Section 2 “Materials and Methods”. The heat treatment was equal for both conditions (annealing at 900 °C for 50 h). The phase-characteristics (content and size) concerning Heusler type phase and γ′-phase were identical for both types of processing techniques and are displayed in Figure 1. Figure 2 shows the grain structures of both states, confirming elongated grains in the direction of load (σ) in the case of directional solidification, while the conventionally cast microstructure exhibits grain boundaries across the orientation of external stress.

**Figure 3:**
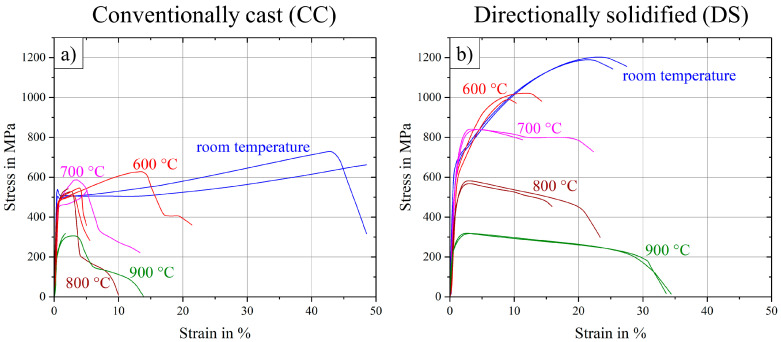
Stress–strain curves at room temperature up to 900 °C for (**a**) conventionally cast samples and (**b**) directionally solidified samples of the base alloy Al_10_Co_25_Cr_8_Fe_15_Ni_36_Ti_6_ in the annealed state (900 °C/50 h).

Results of tensile tests, carried out over a temperature range from room temperature to 900 °C, are displayed in Figure 3. Directional solidification (DS) showed an improvement in two ways as compared to conventional casting (CC). On one hand, DS-samples scattered much less, as standard deviation of particularly ultimate tensile strength (UTS) and also strain to failure (ε_f_) were reduced strongly for each temperature level, see also Table 2. While the deviation is 50 MPa and 8% respectively in CC-state at the most, the maximum deviation in the DS-state is only 16 MPa and 5%. Furthermore, curve progressions for DS-samples seem to be much smoother and more like expected, as the strain to failure increases and the ultimate tensile strength decreases from 600 °C to 900 °C. All curves at room temperature exhibit high ductility with strain to failure levels about 25% for DS-samples and up to 50% for CC-samples.

On the other hand, the most important improvement was the increase of UTS in case of the DS-samples by a factor of 1.5–1.8 in the range of room temperature up to 700 °C. Surprisingly, at higher temperatures of 800 and 900 °C the improvement by DS processing was only small (a factor of 1.1 and 1.0 respectively for 800 and 900 °C).

### 3.3. Influence of Refractory Elements on Microstructural Characteristics

**Figure 4:**
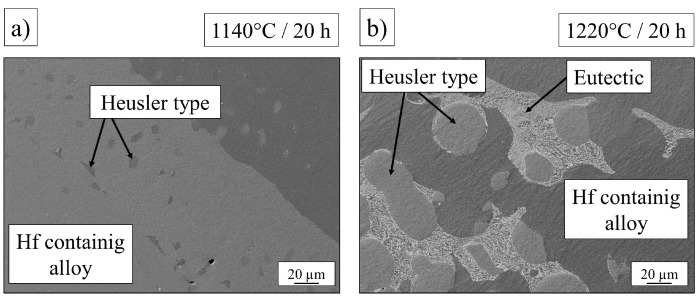
Microstructure of the Hf-containing alloy Al_9.5_Co_25_Cr_8_Fe_15_Ni_36_Ti_6_Hf_0.5_ after homogenization at (**a**) 1140 °C/20 h and (**b**) 1220 °C/20 h.

The original heat treatment of the base alloy has been investigated and adapted for the alloys containing small amounts of hafnium and molybdenum: The homogenization treatment for the base alloy (1220 °C/20 h) was supposed to work for Al_9_Co_25_Cr_8_Fe_15_Ni_36_Ti_6_Mo_1_ and the Hf-containing alloy as well. In the case of the Mo-alloy the Heusler type phase was completely dissolved after this treatment, while the Hf-alloy exhibited eutectic formations at the grain boundaries and unsolved Heusler type phase in spherical form, attached to the grain boundaries between eutectic regions. In this case the heat treatment temperature needed to be adapted and finally no eutectic formation occurred at 1140 °C, but the Heusler type phase still remained unsolved in the homogenized state. These two phenomena are shown in Figure 4.

**Figure 5:**
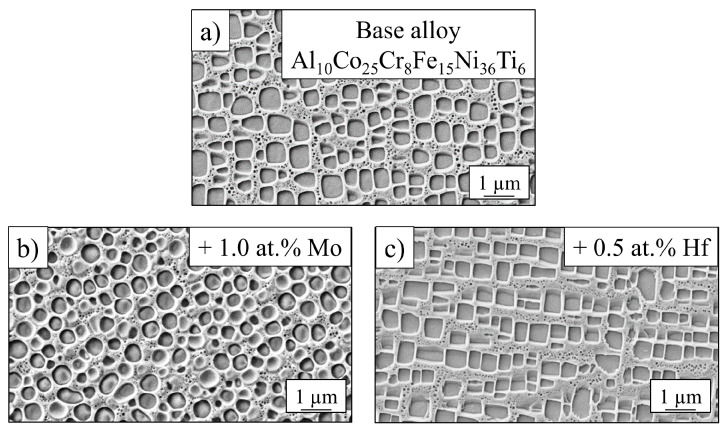
γ′-microstructure of the base alloy (**a**) and with the addition of molybdenum (**b**) and hafnium (**c**) after annealing at 900 °C/50 h.

The annealing step 900 °C/50 h was taken from the initial heat treatment, where γ´-particles precipitate in the fcc-matrix. The γ′-morphology of all alloys after standard treatment is shown in Figure 5 with clear changes: While the base alloy showed cuboidal particles with rounded corners, the Mo-alloy exhibited spherical, and the Hf-alloy showed cubic, sharp-cornered precipitates. These geometries were due to different values of misfit between the γ′-phase and the matrix. It was a result of the differences in lattice parameters of both phases [23]. Experiments for quantitative determination of lattice parameters and therefore misfit values have been carried out with synchrotron radiation at photon source BESSY II in Berlin, Germany over a wide temperature range and are in the process of being evaluated.

**Figure 6:**
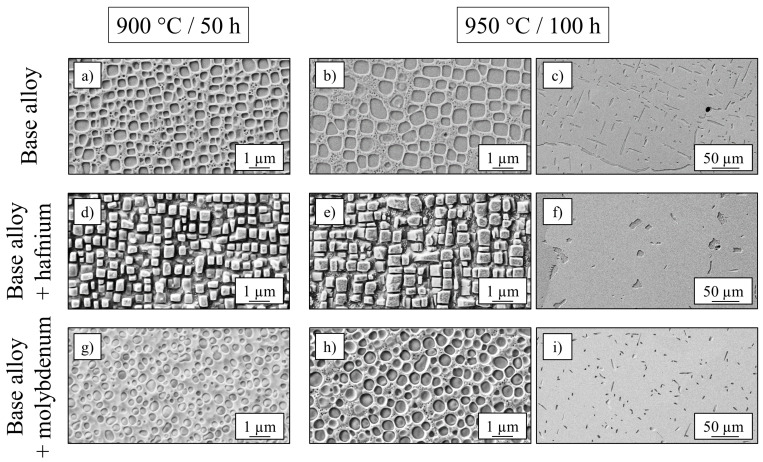
SEM-images showing the γ′- and Heusler type morphology for three different alloys and two different annealing treatments 900 °C/50 h (**a**,**d**,**g**) and 950 °C/100 h (**b**,**c**,**e**,**f**,**h**,**i**).

Nickel-based superalloys with extraordinary strength- and creep-properties at high temperatures exhibited more cubic precipitates, a higher γ′-volume content (60–70%) and larger γ′-particles (up to 500 nm) [24] than the base alloy Al_10_Co_25_Cr_8_Fe_15_Ni_36_Ti_6_ (Vγ′= 40%, dγ′ = 400 nm) after heat treatment at 900 °C/50 h. Therefore, several studies with variation of annealing time and temperature (±50 K; +500 h) have been conducted, resulting in an enhancement of both size and volume fraction after an annealing treatment at 950 °C for 100 h for the base alloy, already described in [25]. SEM-images of both conditions for the base alloy, as well as for Al_9.5_Co_25_Cr_8_Fe_15_Ni_36_Ti_6_Hf_0.5_ and Al_9_Co_25_Cr_8_Fe_15_Ni_36_Ti_6_Mo_1_ are shown in Figure 6, where larger cubic particles in (b), (e) and larger spherical particles in (h) can be detected compared to their original appearance in (a), (d) and (g) after shorter annealing at a lower temperature. Figure 6f shows the spherical Heusler type phase accumulation in the case of the Hf-containing alloy, while the needle-shaped Heusler type phase in the case of the base alloy and the Mo-containing alloy is represented in (c) and (i).

Since volume fraction and precipitate size play an important role for the mechanical behavior, all samples were investigated carefully after both annealing steps using SEM-images. The resulting volume fractions of the γ′- and Heusler type phase, as well as the size of γ′-particles are listed in Table 3. The size corresponds to the diameter in the case of round particles and to the edge-length in case of cuboidal particles.

An increase of γ′-size and γ′-volume fraction, as well as an increase of Heusler type volume fraction is confirmed by Table 3 in the case of the base alloy for the annealing step 950 °C/100 h. While the size of precipitates increased with longer treatment at higher temperatures for the Mo- and Hf-containing alloys, too, the volume fractions of the Heusler type phase and γ′-phase were not enhanced, but decreased about 7–8% in the case of the γ′-phase and remained constant in the case of the Heusler type phase.

### 3.4. High-Temperature Tensile Tests of the Alloys after Annealing at 950 °C for 100 h

**Figure 7:**
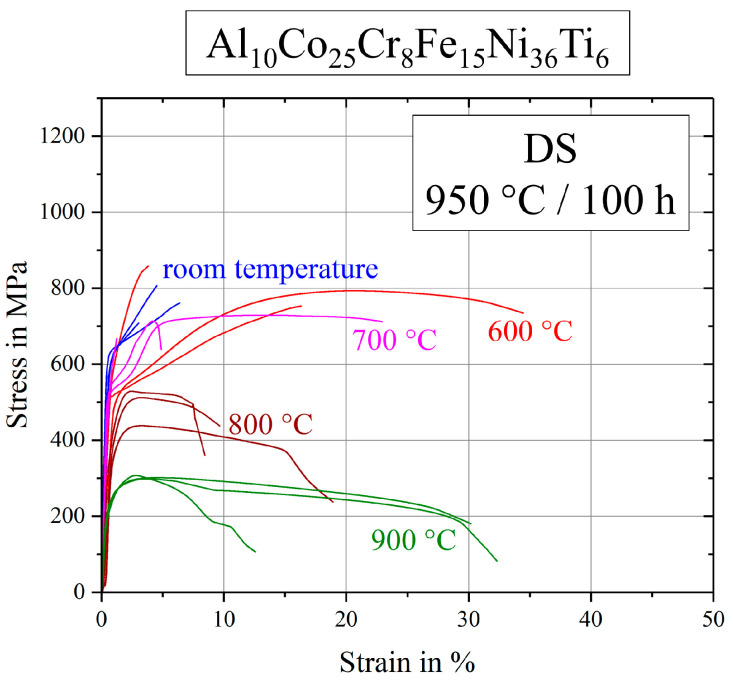
Stress–strain curves for the base alloy Al_10_Co_25_Cr_8_Fe_15_Ni_36_Ti_6_ after annealing at 950 °C for 100 h.

The presented stress–strain diagrams of the base alloy after standard heat-treatment (900 °C/50 h) in Figure 3 are completed by Figure 7, where samples were annealed at 950 °C for 100 h, resulting in larger γ′-precipitates. Samples tested at room temperature showed a brittle fracture behavior, as well as specimens tested at 600 and 700 °C. In general, large scattering occurred respective to strain to failure and curve progression. Ultimate tensile strength scattered less within one temperature series.

**Figure 8:**
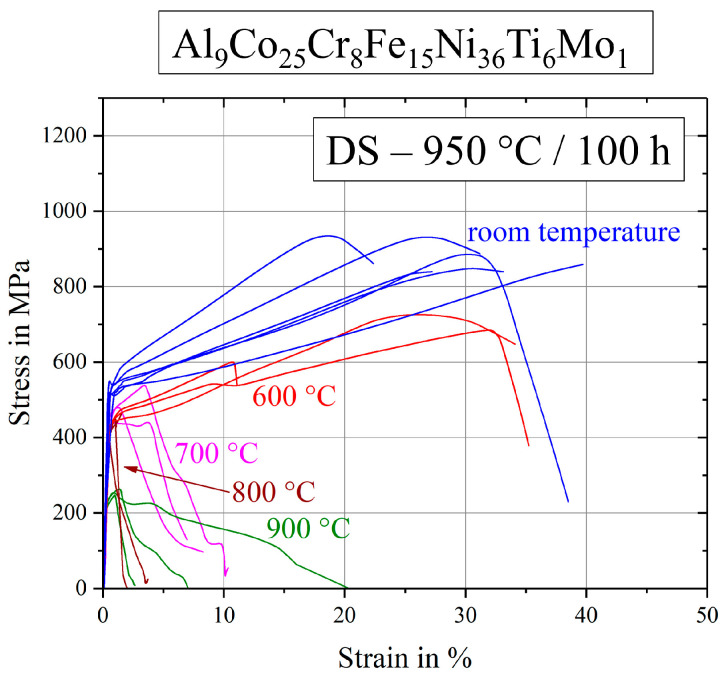
Stress–strain curves for the Mo-containing alloy Al_9_Co_25_Cr_8_Fe_15_Ni_36_Ti_6_Mo_1_ after annealing at 950 °C for 100 h.

Figure 8 shows the stress–strain curves of Al_9_Co_25_Cr_8_Fe_15_Ni_36_Ti_6_Mo_1_ after the annealing step 950 °C/100 h in detail. Scattering of the curve progressions was very high and particularly strain to failure values show large scatter at all temperatures tested. At room temperature and at 600 °C the alloy exhibited a ductile behavior, while the ultimate tensile strength at higher temperatures was reached very quickly after short elongations. In these cases, the first cracks appeared rapidly and fracture propagation was fast.

**Figure 9:**
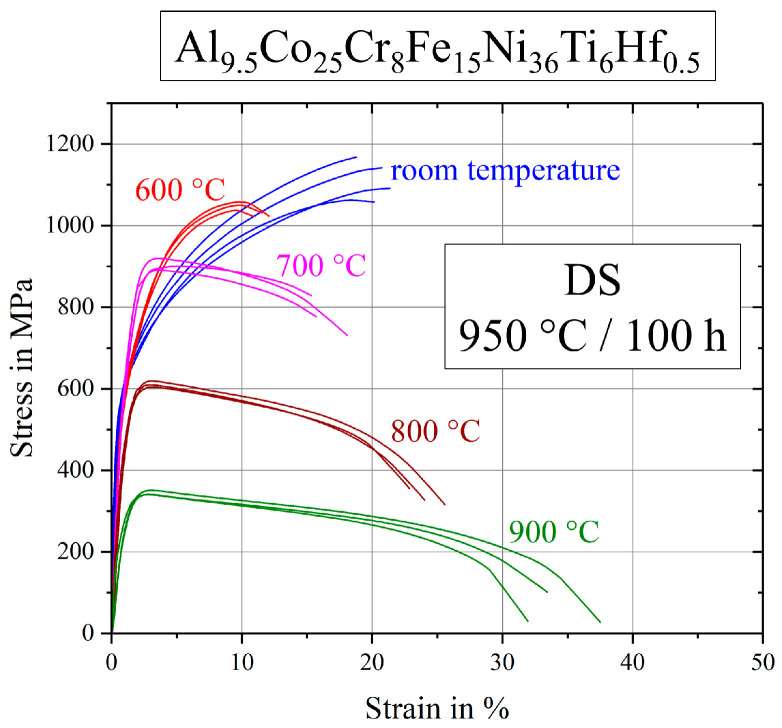
Stress–strain curves for the Hf-containing alloy Al_9.5_Co_25_Cr_8_Fe_15_Ni_36_Ti_6_Hf_0.5_ after annealing at 950 °C for 100 h.

Stress-strain curves of the hafnium containing alloy are displayed in Figure 9. First of all, various tests at the same temperature were very reproducible with only a little scatter. Tests at room temperature reach the highest ultimate tensile strength with a strain to failure of about 20%. Samples deformed at 600 °C exhibited only half the strain to failure. In the range of 600–900 °C the samples showed the expected evolution of strength and plasticity, as ultimate tensile strength decreased and strain to failure increased with increasing temperature.

### 3.5. Discussion and Comparison of Mechanical Properties

In this work, compositionally complex alloys were improved with the goal to increase mechanical properties in the temperature range around 700–800 °C. Therefore, discussion will mainly depend on this temperature region.

**Figure 10:**
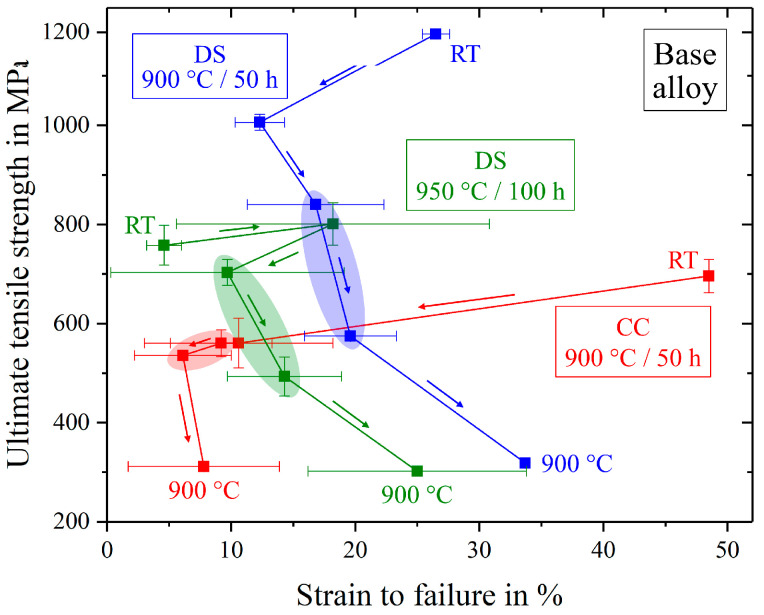
Evolution of ultimate tensile strength and strain to failure from room temperature (RT) to 900 °C for different manufactured and annealed types of the base alloy Al_10_Co_25_Cr_8_Fe_15_Ni_36_Ti_6_.

Figure 10 shows the comparison between different variants of the base alloy respective ultimate tensile strength and strain to failure. The progress of this values from low to high temperatures is indicated by arrows and the significant temperature ranges are marked by differently colored areas. While the polycrystalline samples showed little strength and ductility in the marked red area, both parameters increased using directional solidification (green and blue areas). The two colored areas (green, blue) for directional solidified alloys, with different annealing treatments in contrast, revealed a clear difference: Annealing at 900 °C/50 h did in fact lead to smaller precipitates, but these samples exhibited a higher ultimate tensile strength, higher strain to failure and less scatter. The reason for this desirable behavior was due to the volume content of Heusler type phase that was drastically reduced from 9% to 3% after annealing at 900 °C/50 h, see Table 3.

**Figure 11:**
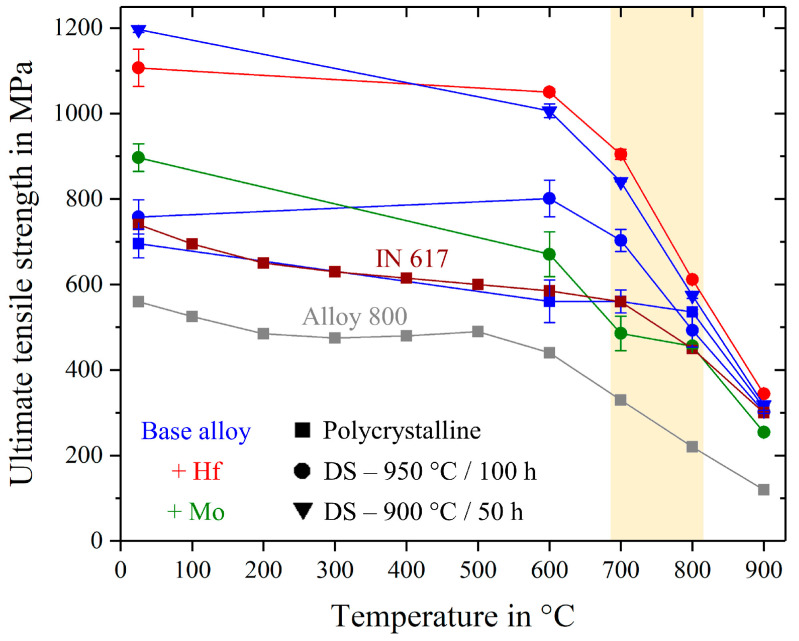
Ultimate tensile strength for all investigated alloys over the temperature range from room temperature to 900 °C. Inconel 617 and Alloy 800 H in a polycrystalline state taken from references [26,27].

An overview about all tested tensile samples is shown in Figure 11, including the different types of the base alloy (blue), the Hf- and Mo-containing alloys in red and green respectively, as well as two conventionally used nickel-based alloys that are used in the temperature-range of 680–820°C, highlighted by the vertical yellow stripe.

**Figure 12:**
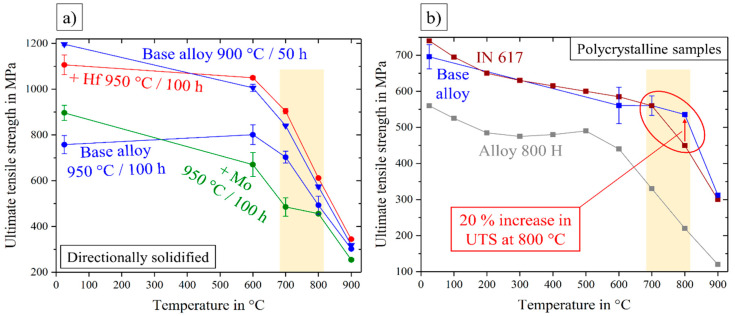
Ultimate tensile strength over the temperature range from room temperature to 900 °C for all directionally solidified alloys (**a**) and the conventionally cast, polycrystalline base alloy, compared with the two commercial nickel-based alloys Inconel 617 [26] and Alloy 800 H [27] (**b**).

Figure 12 shows more detailed views on two types of comparison and allows viewing of the effective aspects of mechanical behavior. Directionally solidified samples were produced to neglect the huge factor of grain-structure and grain-size in the mechanical behavior and to investigate the pure microstructure influence independently.

Thus Figure 12a compares all DS-samples and the base alloy even after two different annealing treatments, leading to the following remarks: Differences in UTS of all tested specimen were getting smaller in the higher temperature range 800–900 °C. Between 700 and 800 °C, and also at 600 °C, there was a clear order: The worst behavior was exhibited by the Mo-containing alloy, the base alloy had a remarkably higher strength and the best mechanical properties could be observed in the case of the Hf-containing alloy. Next to the alloys, annealed under the same conditions, the base alloy after a treatment at 900 °C/50 h was observed in the range of the Hf-containing alloy. Since the base and the Mo-containing alloy (950 °C/50 h) showed the same characteristics of the Heusler type phase, round γ′-particles contributed in a bad way to the mechanical behavior. As the two strongest materials, the base alloy (900 °C/50 h) and the Hf-containing alloy (950 °C/100 h) exhibited almost identical stress–strain diagrams concerning strength, ductility and reproducibility, the small difference in γ′-morphology (sharp corners at the Hf-containing alloy), as well as the shape of Heusler type phase were not a reason for the worse fracture behavior. Consequently, good high-temperature tensile properties occurred in the case of cubic precipitates, assumed that the Heusler type phase content was kept quite low. If the Heusler type phase content rose three times higher in the case of the base alloy, the ultimate tensile strength fell down drastically.

For a comparison with commercially used alloys, the polycrystalline base alloy could be used, see Figure 12b: While the base alloy showed UTS exceeding that of Alloy 800 H over the whole temperature range, it was very similar or only a little worse than the UTS of the alloy Inconel 617 at temperatures up to 700 °C. In the important temperature range between 700 and 800 °C, UTS of the base alloy was equal in the beginning and even exceeded IN 617 at 800 °C by a factor of 1.2.

**Figure 13:**
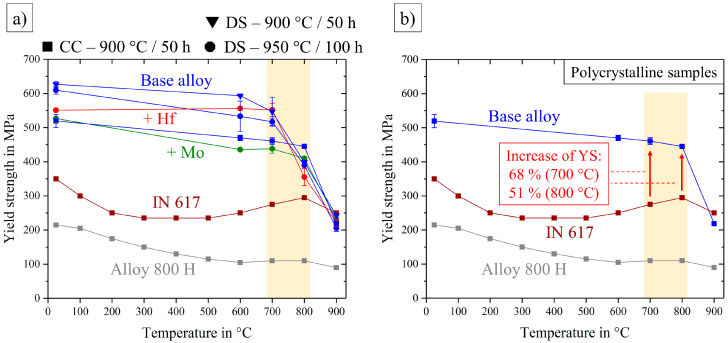
Yield strength over the temperature range from room temperature to 900 °C for all alloys (**a**) and the conventionally cast, polycrystalline base alloy, compared with two commercial nickel-based alloys Inconel 617 [26] and Alloy 800 H [27] (**b**).

Figure 13a shows the yield strength of all tested alloys over the temperature range from room temperature to 900 °C, where the samples showed a remaining plastic deformation of 0.2%. An interesting and application oriented fact is shown in Figure 13b, where the polycrystalline base alloy was compared to the conventionally nickel-based alloys. Similar to the ultimate tensile strength in Figure 12 the yield strength of the base alloy exceeded Alloy 800 H quite significantly. The more competitive alloy IN 617, however, showed similar values of UTS, except at 800 °C, but the yield strength was not able to reach the levels of the base alloy. In the important temperature range the yield strength of the base alloy overran IN 617 by a factor of about 1.7 at 700 °C and 1.5 at 800 °C.

## 4. Conclusions and Outlook

Summarizing the base alloy Al_10_Co_25_Cr_8_Fe_15_Ni_36_Ti_6_ and the influence of Hf (0.5 at.%) and Mo (1.0 at.%) additions on microstructural and mechanical properties:

1. All alloys showed a remarkably high UTS up to 1.26 GPa at room temperature.
2. Casting by the Bridgman process lead to a directionally solidified microstructure and an increase of strength in the direction of the grains by a factor of 1.7 at RT and 1.8 at 600 °C.
3. Addition of 1 at.% molybdenum lead to round γ′-particles, while the Heusler type phase remained in its needle-like shape.
4. An amount of 0.5 at.% hafnium sharpened the corners of the γ′-particles and lead to a spherical Heusler type phase.
5. The γ′-particle size increased for all alloys by annealing for longer times at higher temperature. Volume fractions of γ′ and particularly Heusler type were only increasing in the case of the base alloy, while the Hf- and Mo-containing alloys showed a decrease of γ′-volume fraction and no obvious changes concerning the Heusler type phase.
6. Under the same heat treatment conditions, Mo addition lowered the ultimate tensile strength of the base alloy due to the more round morphology of γ′-particles. The Hf-containing alloy, however, showed an increase in UTS due to the lower content and/or the spherical shape of Heusler type phase.
7. The annealing treatment of 900 °C/50 h for the base alloy lead to a similar mechanical behavior to that of the Hf-containing alloy, with high strength, good strain to failure and high reproducibility. This is referred to a three times reduction of Heusler type volume fraction.
8. In comparison to the two commercially used nickel-based alloys IN 617 and Alloy 800 H, the polycrystalline base alloy showed better mechanical behavior, particularly in the temperature range 700–800 °C and especially in yield strength.

The top priority for future work is to reduce or maybe even avoid the Heusler type phase and to clarify the question about its role on mechanical behavior. We can state that a spherical shape and a low content of <3% is desirable for good mechanical properties. Thus it is necessary to gain knowledge about the chemical and thermodynamic stability resulting in an only two-phase microstructure.

## Figures and Tables

**Table 1:** Chemical composition (in at.%) of all occurring phases in the annealed state (900 °C for 50 h) of the alloy Al_10_Co_25_Cr_8_Fe_15_Ni_36_Ti_6_ analyzed by TEM/EDS [10].

Element Content in at.%	γ-Matrix	γ′-Particles	Heusler Type Phase
**Al**	6.9 ± 0.6	11.4 ± 0.6	24.4 ± 1.3
**Co**	29.5 ± 0.5	22.5 ± 0.6	21.9 ± 1.7
**Cr**	9.3 ± 0.4	3.5 ± 0.4	3.6 ± 0.2
**Fe**	20.4 ± 0.6	8.8 ± 0.7	10.7 ± 0.4
**Ni**	30.4 ± 1.0	45.0 ± 1.5	33.9 ± 0.6
**Ti**	3.5 ± 0.4	8.7 ± 0.5	5.6 ± 0.2

**Table 2:** Ultimate tensile strength (UTS) and strain to failure (ε_f_) at different temperature levels for the conventionally cast (CC) state and the directionally solidified (DS) state.

	UTS in MPa		YS in MPa		ε_f_ in %	
	CC	DS	CC	DS	CC	DS
**~23 °C**	696 ± 33	1197 ± 6	520 ± 19	627 ± 9	49 ± 0	27 ± 1
**600 °C**	561 ± 50	1006 ± 16	470 ± 8	594 ± 3	11 ± 8	12 ± 2
**700 °C**	560 ± 27	840 ± 1	461 ± 10	547 ± 42	9 ± 4	17 ± 5
**800 °C**	536 ± 7	575 ± 7	445 ± 2	399 ± 1	6 ± 4	20 ± 4
**900 °C**	312 ± 6	319 ± 1	219 ± 6	243 ± 2	8 ± 6	34 ± 1

**Table 3:** Volume fractions of γ′- and Heusler type phase, size and shape of γ′-precipitates after the annealing treatments 900 °C/50 h and 950 °C/100 h.

		Base Alloy	Base Alloy +Mo	Base Alloy +Hf
900 °C 50 h	d_γ′_ in nm	200 ± 70	190 ± 70	210 ± 70
	V_γ′_ in %	38 ± 7	44 ± 1	46 ± 2
	V_Heusler_ in %	3 ± 2	3 ± 3	6 ± 1
950 °C 100 h	d_γ′_ in nm	400 ± 100	360 ± 100	420 ± 100
	V_γ′_ in %	41 ± 3	37 ± 1	38 ± 8
	V_Heusler_ in %	9 ± 1	3 ± 1	5 ± 1
Shape of γ′-precipitates		Cuboidal (round corners)	Round	Cuboidal (sharp corners)
